# SARS-CoV-2 Exploits Non-Canonical Autophagic Processes to Replicate, Mature, and Egress the Infected Vero E6 Cells

**DOI:** 10.3390/pathogens11121535

**Published:** 2022-12-14

**Authors:** Juraj Koči, Marta Novotová, Monika Sláviková, Boris Klempa, Ivan Zahradník

**Affiliations:** 1 Institute of Virology, Biomedical Research Center, Slovak Academy of Sciences, 84505 Bratislava, Slovakia; 2 Institute of Experimental Endocrinology, Biomedical Research Center, Slovak Academy of Sciences, 84505 Bratislava, Slovakia; 3 Department of Microbiology and Virology, Faculty of Natural Sciences, Comenius University in Bratislava, 84215 Bratislava, Slovakia

**Keywords:** SARS-CoV-2, autophagy, mitophagy, virus maturation, virus egress, gene regulation

## Abstract

The coronavirus transforms the cytoplasm of susceptible cells to support virus replication. It also activates autophagy-like processes, the role of which is not well understood. Here, we studied SARS-CoV-2-infected Vero E6 cells using transmission electron microscopy and autophagy PCR array. After 6–24 h post-infection (hpi), the cytoplasm of infected cells only contained double-membrane vesicles, phagophores, and phagosomes engulfing virus particles and cytoplasmic debris, including damaged mitochondria. The phagosomes interacted with the viral nucleoprotein complex, virus particles, mitochondria, and lipid droplets. The phagosomes transformed into egress vacuoles, which broke through the plasmalemma and discharged the virus particles. The Vero E6 cells exhibited pronounced virus replication at 6 hpi, which stabilized at 18–24 hpi at a high level. The autophagy PCR array tests revealed a significant upregulation of 10 and downregulation of 8 autophagic gene markers out of 84. Altogether, these results underline the importance of autophagy-like processes for SARS-CoV-2 maturation and egress, and point to deviations from a canonical autophagy response.

## 1. Introduction

Several cellular mechanisms involved in coronavirus infection, including that caused by the severe acute respiratory syndrome coronavirus 2 (SARS-CoV-2), have been described down to the molecular level [1,2,3]. The entry of the virus begins as the viral spike protein binds to the receptor angiotensin I, converting enzyme 2 (ACE2) via its receptor binding domains, and it is primed by cellular proteases to initiate the membranes’ fusion [4,5]. Recent studies suggest that coronaviruses replicate in the cytoplasm by hijacking cellular RNA and protein synthesis systems [6,7]. Coronaviruses target the two major organelles involved in cellular proteosynthesis, endoplasmic reticulum (ER) and Golgi apparatus (GA), causing the disintegration of the ER network and fragmentation of the GA [8,9]. Despite the intensive research, the maturation and egress of the virus are less understood due to the complexity of the process and ambiguous findings. The formation of coronavirus particles begins when a sufficient amount of viral RNA and protein components have been synthesized. The virus ribonucleoprotein complex (vRNP) starts to cluster at the surface of specific double-membrane vesicles (DMVs) of the endoplasmic reticulum and Golgi intermediate compartment (ERGIC). By budding through the vesicle membrane to the interior of the vesicles [2], the virus particles gain their membrane and associated proteins and prepare for maturation. This process is paralleled by the formation of diverse vesicles and complex membrane bodies originating from ERGIC, which contain viruses at different stages of maturation [10,11]. Lysosomes, organelles directly involved in the autophagic pathway, also play a role in coronavirus trafficking and exocytosis of mature virions [12,13]. Finally, the virus-containing vesicles transform, fuse with the plasmalemma, and release mature viruses in the extracellular space [11].

The morphologic and molecular evidence revealed activation of the autophagic process by various RNA viruses as well as coronaviruses to promote their replication [14,15,16]. Autophagy, as a constitutive pathway activated in cells during starvation or infection, involves the generation of double-membrane vesicles called autophagosomes. It is important to note that mitochondria are degraded by autophagy (mitophagy) too. Mitophagy can also be triggered by viruses binding to mitochondrial receptors to evade innate immunity by promoting sequestration into a phagophore [17,18]. According to the canonical process, autophagosomes form from phagophores (appearing in the electron microscope as flattened membrane vesicles) by growing the membrane and engulfing a part of the cytoplasmic volume with remnants of damaged organelles, protein aggregates, and pathogens [19]. Once fully enclosed, a phagophore fuses with an acidic lysosome providing proteases, which digest the autophagosomal content before its release to the extracellular space [19]. Formation of autophagy-specific phagophores requires the activation of the phosphatidylinositol 3-kinase complex containing protein PIK3C3, BECN1, and other autophagy-related proteins (Atg) (Atg38, Atg14, BECN1/Atg6). Phagophore growth and maturation occur via the action of the ubiquitin conjugation system (ULK complex) regulated by the mammalian target of rapamycin (mTOR) and the activity of several other Atg8-family proteins such as LC3 and GABARAP [15,20]. Activation and a positive role of autophagy were demonstrated in the case of various coronaviruses including SARS-CoV, although activation of the full pathway was not required for virus replication [21]. In addition, there is strong experimental evidence that SARS-CoV replication is independent of a functional ubiquitin–proteasome system (UPS) [22] and that autophagy pathways may not be directly implicated in the replication of the virus, unlike for other coronaviruses [23]. Moreover, as recent findings show, autophagy may also be negatively regulated by the MERS coronavirus to promote its replication [24]. Therefore, questions about whether and how the cellular autophagosomal process is involved in SARS-CoV-2 replication, assembly, and egress warrants further investigation.

In this study, we analyzed the transformation of the cytoplasm of Vero E6 cells infected by the SARS-CoV-2 isolate. We focused on autophagy-like processes activated by virus replication by using transmission electron microscopy and PCR array tests for autophagy-related genes. We found morphological support for the activation of the autophagy but deviated from the canonical processes, which was supported by the partial regulation of autophagy-related genes.

## 2. Materials and Methods

### 2.1. Cell Cultures and Infection with SARS-CoV-2

Vero E6 cells (Vero C1008, ATCC CRL 1586) were purchased from ATCC, Manassas, USA, and cultured in a T-25 cell culture flask (Greiner Bio-One, Frickenhausen, Germany) as adherent monolayer until ~80% confluence in DMEM culture medium (Dulbecco’s Modified Eagle’s Medium), supplemented with 5% fetal bovine serum, antibiotics penicillin-streptomycin (Gibco ThermoFisher Scientific, Waltham, MA, USA), and the antimycotic amphotericin B (MilliporeSigma, Darmstadt, Germany) at 37 °C and 5% CO_2_ saturation. This cell line was chosen as it supports high levels of SARS-CoV-2 replication and propagation [25]. Cells were infected with SARS-CoV-2, strain Slovakia/SK-BMC5/2020. The strain was isolated from a COVID-19 patient from Slovakia in March 2020, and the complete sequence was deposited on GISAID.org under the accession number ID EPI_ISL_417879. The isolate was deposited in the European Virus Archive GLOBAL and is available at https://www.european-virus-archive.com/virus/sars-cov-2-strain-slovakiask-bmc52020 (accessed on 6 March 2020). Three hundred µl of the virus suspension (titer 3 × 10^7^/mL) was added to the cells and incubated for 1 h at 37 °C and 5% CO_2_ saturation. The virus was removed from cell cultures by washing with sterile buffer solution pH 7.2. Infected cells were further incubated in a fresh culture medium for 6, 18, and 24 h. Non-infected cells were prepared and processed in parallel as a negative control.

### 2.2. SARS-CoV-2 Replication In Vitro and TCID_50_ Assay

The replication dynamic of SARS-CoV-2 in Vero E6 cells infected at specific time points post-infection (hpi) as specified above was measured using quantitative PCR (qPCR). Total RNA from the infected and control cells was isolated using Trizol Reagent (ThermoFisher Scientific, Waltham, MA, USA). One µg of total RNA was reversely transcribed to cDNA using the LunaScript RT Supermix kit, employing oligo dT/random hexamer protocol (NEB, Ipswich, MA, USA). One µL of cDNA was used in SYBR Green-labelled PCR reaction of which parameters were as follows: initial denaturation at 95 °C for 10 min, followed by 40 cycles at 95 °C for 10 s, and 58 °C for 1 min. The final step in the amplification cycle was a melt curve analysis at 65 °C for 30 s, increased by 0.5 °C per cycle to 95 °C, to ensure the specificity of the amplicons. The amplification was performed in an AriaMx real-time thermal cycler (Agilent Technologies, Santa Clara, CA, USA) using Maxima SYBR Green ROX master mix (Thermo Fisher Scientific, Waltham, MA, USA). Viral load in the cells was based on a transcript level of the E gene, detected with the following primers F: 5′-ACA GGT ACG TTA ATA GTT AAT AGC GT-3′; R: 5′-ATA TTG CAG CAG TAC GCA CAC A-3′ [26]. It was normalized with a house-keeping gene, β-actin, using following primers F: 5’-TCC TCC CTG GAG AAG AGC TA-3′; R: 5′-ACA TCT GCT GGA AGG TGG AC-3′.

TCID_50_ assay was performed to estimate the sensitivity of the studied cell line to the virus extract (24 hpi) and visualize its cytopathic effect. A serially diluted virus medium from 10^−1^ to 10^−10^ was added to subconfluent cells growing in 96-well plates in quadruplicates and incubated for 4 days at 37 °C and 5% CO_2_. Following a 4-day incubation, the cell culture medium containing detached cells was removed, and the remaining attached viable cells were fixed with 4% formaldehyde for 20 min at room temperature (RT). After fixation, cells were stained with crystal violet for visualization.

### 2.3. Transmission Electron Microscopy

Upon 6, 18, or 24 h post-infection, the culture medium with SARS-CoV-2 was discarded, and the cells were washed with sterile buffer solution pH 7.2 and enzymatically detached from the culture flask using TrypLE Express reagent (Gibco ThermoFisher Scientific, Waltham, MA, USA). Detached cells were pelleted at 200 g for 10 min, re-suspended and washed in sterile buffer solution pH 7.2, and centrifuged at 200 g for 10 min. Cell pellets were fixed in 2% glutaraldehyde in cacodylate buffer (150 mM Na-cacodylate, 2.0 mM CaCl_2_ at pH 7.3). After a brief prewash with the fixative buffer, the pellets were prefixed in a fresh aliquot of the fixative for 2 h at 4 °C in centrifuge tubes. The hardened pellets were transferred to glass vials containing a fresh fixative and incubated again for 2 h at 4 °C. Upon fixation, the pellets were continuously washed with cacodylate buffer for 10 min, post-fixed for 45 min in 1% osmium tetroxide (OsO_4_) in cacodylate buffer, contrasted overnight in the saturated aqueous solution of uranyl acetate at RT, dehydrated in graded ethanol series and propylene oxide, embedded into Durcupan (Fluka, AG, Buchs, Switzerland), and polymerized at 60 °C for 3 days. Ultrathin sections (58–60 nm) were cut by Power-Tome MT-XL (RMC/Sorvall, Tuscon, AZ, USA) ultramicrotome, placed on formvar-coated copper grids, and contrasted with lead citrate. Ultrathin sections were examined with JEM 1200 electron microscope (Jeol, Tokyo, Japan) at 80 kV. Selected cell images were recorded by the Gatan Dual Vision 300 W CCD camera (Gatan Inc., Pleasanton, CA, USA) at a magnification of 30,000–200,000×. Due to comparable morphological observations in cells inspected at all post-infection time points, we present herein only cell images taken at 24 hpi.

### 2.4. RT^2^ Profiler Autophagy PCR Array in SARS-CoV-2-Infected Cells

An autophagy PCR array was carried out in Vero E6 cells sampled at 24 hpi to correlate with the microscopic observations. Upon RNA isolation as described above, the RNA concentration and quality were assessed using the NanoDrop spectrophotometer (NanoDrop Technologies LLC, Wilmington, DE, USA) and RNA LabChip BioAnalyzer (Agilent Technologies, Santa Clara, CA, USA), respectively. To ensure reliable results in subsequent PCR arrays, only RNA samples with a RIN number above 7 were used. Five µg of total RNA was reversely transcribed to cDNA using the RT^2^ First Strand kit (Qiagen, Hilden, Germany), employing the genomic DNA elimination buffer and the oligo dT/random hexamer protocol. The RT^2^ Profiler Autophagy PCR array (Qiagen, Hilden, Germany) was used for the expression of the multiple autophagy-related genes potentially activated in response to SARS-CoV-2 infection. One array in 96-well format per sample (control and experimental) was used according to the manufacturer’s manual. Raw expression data (C_q_ values) were converted into the fold-change/regulation values in the data analysis web-based tool available at SABiosciences.com.

## 3. Results and Discussion

### 3.1. Morphology of Infected Cells

**Figure 1:**
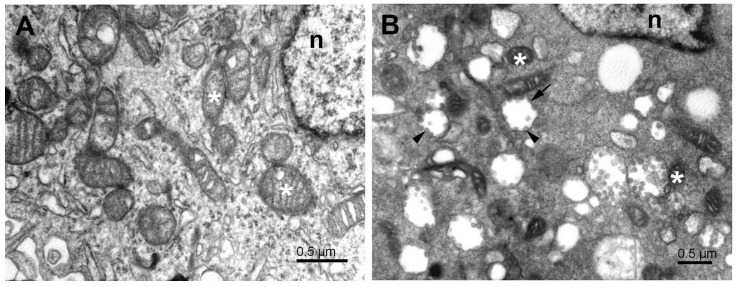
Subcellular structures of the cell lines infected with SARS-CoV-2. (**A**) Typical electron microscopy image of control Vero E6 cell showing cytoplasm with organelles near the nucleus. (**B**) Typical EM image of cytoplasm near the nucleus of SARS-CoV-2-infected Vero E6 cell 24 h post-infection. White asterisks—mitochondria, n—nucleus, short arrow—vesicle with attached virus particles, arrowheads—virus particles.

The transformation of Vero E6 cells infected with SARS-CoV-2 was studied using transmission electron microscopy to characterize cellular details during virus maturation up to the egress. The major effect of the post-infection time was in the relative number of normal, infected, and dead cells in the culture dish rather than in the morphology of the infected cells. This indicates that once the cell was infected, it underwent the morphological transformation relatively fast. As the exact time of infection of individual cells was not known, we focused here on general changes. The overall features that characterized infected cells (Figure 1) included large regions of electron-dense cytoplasm containing numerous vesicles and membrane bodies, modified and split mitochondria, lipid droplets, numerous ribosomes, emerging phagophore, phagosome-like structures, and numerous virus-like particles. Endoplasmic reticulum and Golgi apparatus membranes, with morphology as in naïve cells, were rare.

**Figure 2:**
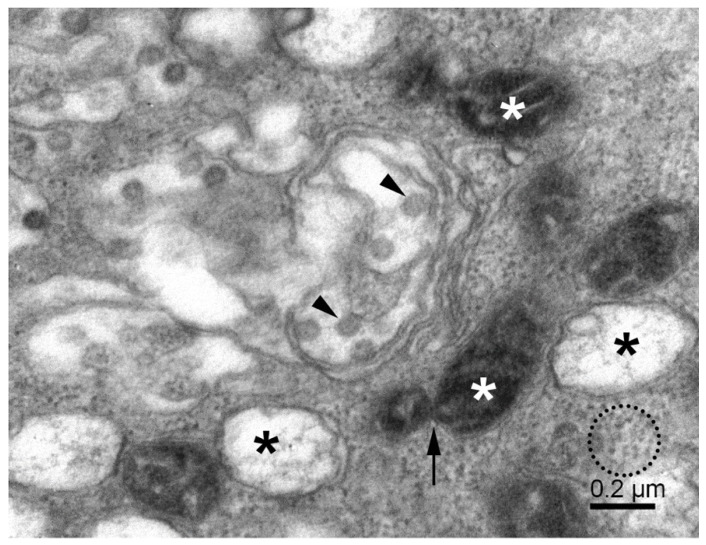
Replication of SARS-CoV-2 in Vero E6 cells. The cytoplasmic area shows various membrane vesicles (known as ERGIC) with virus particles (arrowheads), double-membrane vesicles with fibrous material (black asterisks), small mitochondria (white asterisks), mitochondrial fission (arrow), and the dense cytosol with freely distributed ribosomes and RNPs (dotted circle).

The higher electron density of cytosol in the infected Vero E6 cells (Figure 1B and Figure 2) resulted to a large extent from the increased amount of freely distributed ribosomes and the intense production of virus constituents. The endoplasmic reticulum was transformed into a system of proliferated membranes of various shapes and sizes. A similar transformation comprised Golgi membranes that, together with proliferating ER, gave rise to copious membrane bodies (Figure 2), as described previously [27]. Of special importance are double-membrane vesicles (DMVs), which were shown to be an essential part of the ER–Golgi intermediate compartment (ERGIC), the site of coronavirus replication [2]. The presence of numerous single and double-membrane vesicles and the appearance of virus-like particles is the universal signature of cells infected by coronaviruses [28,29]. The same is valid for the replication complex of SARS-CoV-2 in the Vero E6 cells. The replication complex is known to provide a structural substrate for assembling virus nucleocapsids with membrane envelopes and de novo formation of the virus particles. This process was described as the budding of the vRNP complex to the lumen of the vesicle through the membrane with an already built-in spike protein complex [30,31]. However, as we will show below, the virus budding could be more complex, as was the fate of the virus particles in the cell from the assemblage, through maturation, up to egress.

### 3.2. Mitochondria, Fission, and Mitophagy

**Figure 3:**
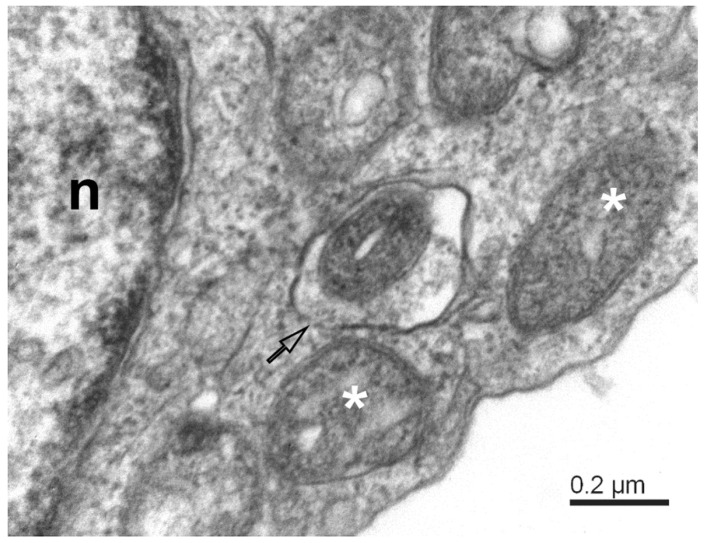
Mitophagy-like structures and degradation of mitochondria in SARS-CoV-2-infected Vero E6 cells. The mitophagosome encapsulates a degrading mitochondrion. Note that the mitophagosome membrane is not fully closed (open arrow); white asterisks—degrading mitochondria; n—nucleus.

**Figure 4:**
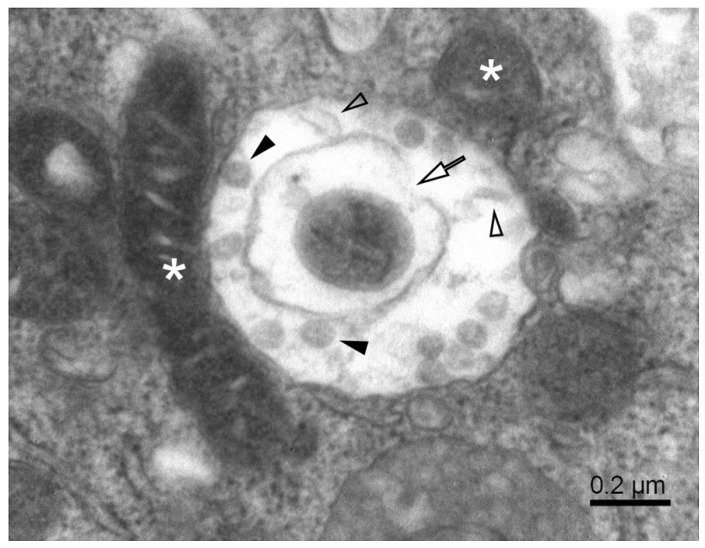
A complex autophagosome in SARS-CoV-2-infected Vero E6 cell. Apposition of mitochondria and a large autophagosome containing an open mitophagosome (open arrow). Open arrowheads—cell debris; solid arrowheads—virus-like particles; white asterisks—mitochondria.

The mitochondrial population in Vero E6 cells was highly heterogeneous, and in infected cells, it was often partially damaged. Small mitochondria resulted from the ongoing process of mitochondrial fission (Figure 2). A large surface-to-volume ratio in small mitochondria might increase the efficiency of energy production. On the other hand, numerous damaged mitochondria point to the loss of energy supply. The damage involved the disintegration of the mitochondrial membranes, both the outer and inner ones. The small and damaged mitochondria were often surrounded by the membrane, resembling the autophagy-like structures typical for mitophagy (Figure 3). In conjunction with excessive mitochondrial splitting, mitophagy indicates mitochondrial stress due to increased levels of reactive oxygen species and the activation of the cellular immune reaction [32]. The infected cells displayed well-developed phagosomes containing some cell debris, virus-like particles, and even a phagosome engulfing a remnant of a mitochondrion. A notable feature was the incomplete enclosure of the phagosomes, which allows the exchange of material with the cytosol. According to canonical autophagy, the double membrane-enclosed phagosomes are expected to merge with acidic lysosomes to digest their content. Interestingly, we have not observed such fusion in infected cells; instead, we could see a different development of open phagosomes (Figure 4).

### 3.3. Phagophore, Phagosomes, and Autophagy

**Figure 5:**
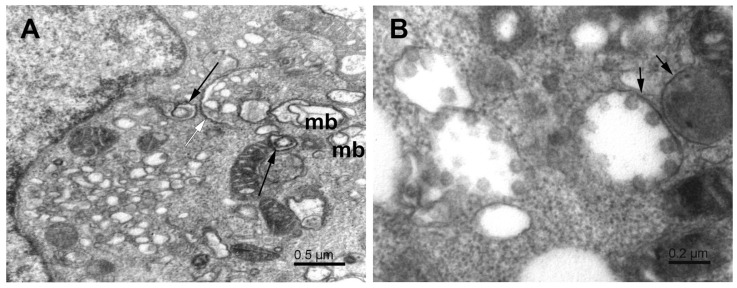
Phagophores in SARS-CoV-2-infected Vero E6 cells. (**A**) Cytoplasm with various phagophores; multivesicular body (mb), double-membrane vesicles (long arrows), and flattened zipped ER membranes (white arrow). (**B**) Phagosome-like vesicles (short arrows) with virus particles. Note that the membranes of these vesicles do not fully close, and the virus particles are mostly bound to the concave surface.

The cytosol of infected cells contained double-membrane structures of various forms. Phagophores appeared as strongly flattened vesicles with partially or fully zipped membranes that showed a tendency to engulf cytoplasmic material (Figure 5A). Other phagophore-like double-membrane vesicles were often visually empty or contained one or a few virus particles (Figure 5B). Interestingly, these vesicles did not fully close to the form of a typical autophagosome. At low magnification, these vesicles looked as if they were enclosed by the membrane; however, a closer inspection at a higher magnification always revealed vesicle segments which were not lined with the membrane. Moreover, the vesicle content was visually clear; that is, its electron density was much lower than that of the surrounding cytosol. This can be explained by the local activity of cytolytic enzymes, but it is not clear how was their activity was limited only to the inner subspace of the vesicle. As the border between the clear lumen and the surrounding cytosol was visually well-defined, we will refer to these vesicles as phagosomes, despite the partial absence of the limiting membrane. Cellular material or viruses were well retained within the phagosome (Figure 5B).

**Figure 6:**
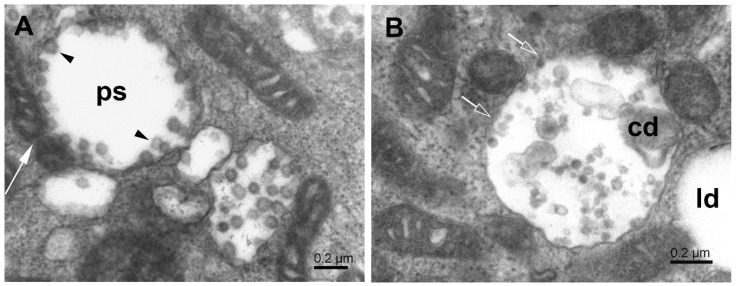
Phagosome formation at mitochondrial surface. (**A**) Phagosome (ps) created near splitting mitochondrion (white long arrow) and enclosing the maturing virus particles (arrowheads). (**B**) The autophagosome, in contact with small mitochondria, contains cell debris and virus-like particles. Open arrows point to sites of missing membrane; cd—cell debris; ld—lipid droplet.

The phagosomes containing virus particles were often in contact with neighbor organelles. This feature is a common attribute for which the endoplasmic reticulum is considered the major source of phagophores [27,33,34,35,36]. We observed the formation of phagophores also near the surface of mitochondria and at sites of mitochondrial fission (Figure 6A,B). Such phagosomes contained virus particles, and thus, their membrane could participate in virus maturation. Interestingly, the phagosomes eventually contained both the virus particles and the cell debris and thus resembled classical autophagosomes (Figure 6B). Except for virus particles, these observations agree with previous studies reporting mitochondrial division occurring concurrently with autophagosome formation [33], phagophore formation from the outer mitochondrial membrane [34], or the generation of phagophores/autophagosomes “de novo” from mitochondrial membranes [35,36]. In those studies, the outer mitochondrial membrane was considered another source of autophagosomal lipids. Our observations of viral particles close to degrading mitochondria (Figure 6B) suggest the participation of mitochondrial membrane in the virus maturation process.

### 3.4. Lipid Droplets

**Figure 7:**
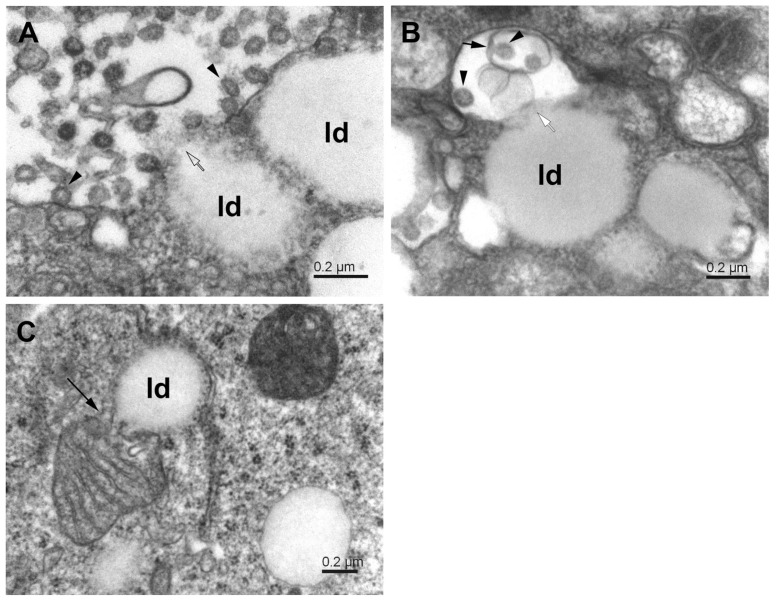
Interaction of phagosomes with lipid droplets in SARS-CoV-2-infected Vero E6 cells. (**A**) Phagosome with virus particles (arrowheads) in close contact with lipid droplets (ld); open arrow—open membrane. (**B**) The lipid droplets (ld) in contact with a phagosome (white arrow), which contains a small phagosome (short arrow) encapsulating virus particles (arrowheads). (**C**) Apposition of mitochondrion and lipid droplet (ld). The long arrow points to the site of mitochondrial membrane degradation.

It was shown that viruses may target lipid droplets to replicate and/or for nutritional and anti-immunity purposes [37]. Lipid droplets are of importance also to phagophore formation [35,38]. We observed the interaction of lipid droplets with phagosomes (Figure 7A,B). Interestingly, the interaction occurred almost exclusively at the open border of the phagosome and with the lipid droplet without the limiting membrane. Phagosomes interacting with lipid droplets contained virus particles and often also some cytoplasmic debris (Figure 7B). Notably, similar interaction was present between lipid droplets and damaged mitochondria (Figure 7C). Both organelles had lost, at least locally, their limiting membrane, which allowed their content to interact directly. The reason and results of such interactions for virus proliferation remain to be clarified. The recent research suggests the involvement of host lipids in the replication of several positive-strand RNA viruses, which manipulate lipid metabolism in the cell to ensure the availability of specific types of lipids [39]. Apposition of the lipid droplets to the site of the newly formed virus underlines the role of lipid droplets in the maturation of SARS-CoV-2 [40].

### 3.5. Virus Assembly, Maturation, and Egress

**Figure 8:**
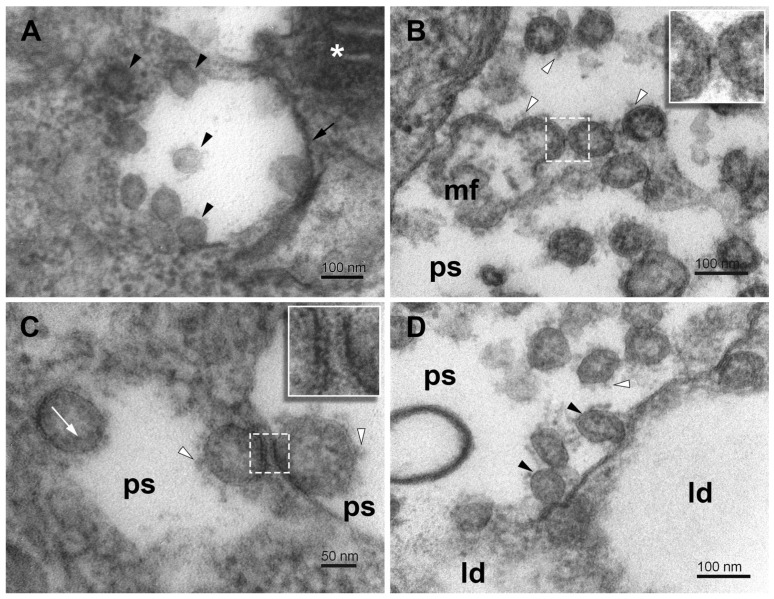
Virus particles in phagosome vesicles in SARS-CoV-2-infected Vero E6 cells. (**A**) Virus particles of different morphology in the phagosome and nearby cytosol (arrowheads). (**B**) A group of budding virus particles in the lumen of a phagosome. Inset—a detail of the stalk between budding virus particles. (**C**) Virus particles adhering to remnants of phagosome membrane. Inset—a detail of the stalks between the virus particle and the phagosome membrane. (**D**) Virus particles in the lumen of the phagosome in direct contact with the lipid droplets. Arrowheads—virus particles; short arrow—phagosome membrane; white arrowheads—spikes; white arrow—nucleocapsids; ps—lumen of phagosomes; mf—a membrane fragment; white asterisk—mitochondrion; ld—lipid droplet.

The synthesized virus particles typically anchor to the inner concave surface of a phagosome lumen (Figure 6A,B and Figure 7B) or membrane remnants in a phagosome (Figure 7B and Figure 8B). According to the budding theory [41], the SARS-CoV virus is completely assembled by the budding of the cluster of vRNP complexes, synthesized in the cytoplasm, through the membrane of specialized vesicles, which are a part of the ERGIC system producing virus proteins, including membrane-associated proteins. Thus, the freshly budded virus particle is almost completely assembled. Before egress, however, the virus particles have to be transported toward the cell surface, in which phagosomes fulfill an indispensable role, as we showed here. The membrane of a phagosome is considered as another cell substrate hijacked by the virus replication machinery for the morphogenesis and maturation of the virus [15,16]. However, the canonical role of phagosomes is in the process of autophagy, used by cells universally to collect and destroy foreign and damaged material in the cytoplasm to keep cells alive. Our observations are not so unequivocal. The virus-like particles inside the phagosomes are of variable morphology. Many particles look like a completely assembled virus including the spikes, but many miss a part of the membrane envelope and display various structural deficiencies (Figure 6 and Figure 8). These observations do not have a simple explanation and do need further research. Nevertheless, the variable morphology indicates that the incomplete virus particles could result from erroneous budding, or more likely, from the damage of the virus by the activity of lytic enzymes in phagosomes, as would be expected for incomplete autophagy. The failed budding is also indicated by the presence of virus particles at the membrane-free border of the phagosome and even in the nearby dense cytosol (Figure 8A). The incomplete autophagy is indicated by the open structure of phagosomes, the deficiency of lysosomes, and the formation of egress vacuoles bearing the matured virus particles out of the cell. However, the presence of phagosomes itself means that the cell autophagy response was activated, although not completed down to virus elimination.

The open structure of phagosomes, observed in this study, allows the interaction of their content with the cytosol, which might influence the virus engulfed in the phagosomes. The virus-like particles occurring at the cytosolic and luminal sides of the membrane-free border indicate that the virus might acquire its membrane also by other than the budding mechanism (Figure 8B). Hypothetically, the virus precursors could enter the phagosome at the open region, adhere to the concave side of the phagosome, and harbor its membrane (Figure 8A,C). Alternatively, the virus precursor that entered a phagosome could make use of an encapsulated membrane fragment. Missing pieces of the phagosome membrane envelope could result from its transfer to virus precursors (Figure 8D). Unfortunately, the resolution of transmission electron microscopy on chemically fixed samples did not allow us to identify spike protein on the phagosome membrane and to be more specific about the ongoing processes. A reverse process, that is, the virus losing its coat in the phagosome should be also considered.

We have not observed the fusion of phagosomes with lysosomes and the formation of canonical autophagosomes. This is in line with recent studies reporting on mechanisms that the viruses have developed for the inhibition of the fusion and the autophagic machinery to support their replication [15,20,21]. Instead, we have observed the formation of the egress vacuoles bearing virus particles, typically attached to their concave surface, close to the cell surface.

**Figure 9:**
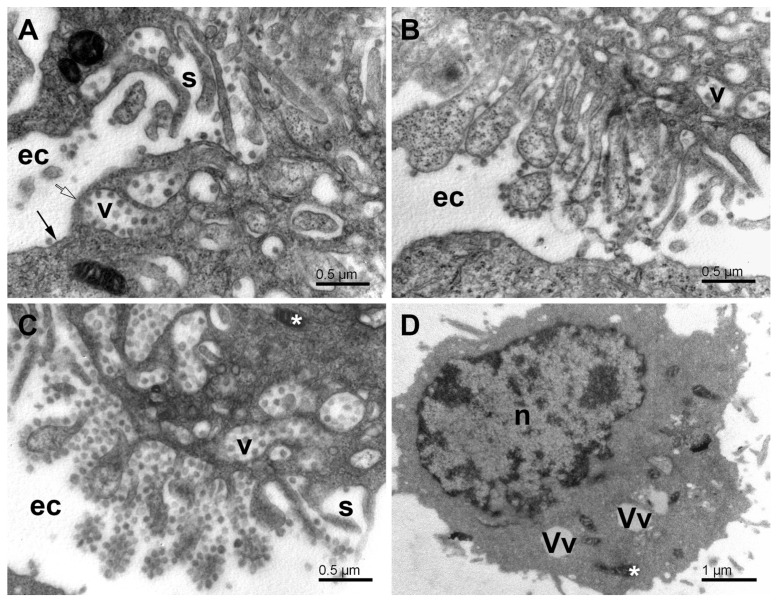
Egress of virus particles from SARS-CoV-2-infected Vero E6 cells. (**A**) Accumulation of the egress vacuoles under the cell surface. Note the virus particles anchored at the concave side of the vacuoles. (**B**) Outer surface of a cell with evaginated egress vacuoles. The virus particles are still anchored to the membrane, but now they are at the convex side of the evaginations. The cell segment at the bottom of the image shows a part of an uninfected cell. (**C**) Virus particles adhering to the evaginated cell membrane and clustered like grapes while exposed to the extracellular milieu. (**D**) The cell after the virus egress. Ec—extracellular space; long arrow—the surface membrane; open arrow—the membrane–free surface area; s—the surface lamellae; v—the egress vacuoles; n-nucleus; Vv—vacuoles; white asterisks—mitochondria.

In the final step, the egress vacuoles reached the plasmalemma. The fusion of egress vacuoles with plasmalemma, such as described for transport vesicles and plasmalemma, was not observed in this study. Instead, we observed local disruption or cracking of plasmalemma and the formation of plasmalemmal lamellae of about 100 nm across, separating the egress vacuoles from the extracellular space (Figure 9A). These lamellae were mostly free of the lipid membrane and locally separated the egress vacuoles from the extracellular space at their membrane-free perimeter. In many places, the lamellae were broken, and thus, the lumen of the egress vacuoles was directly connected to the extracellular space. Nevertheless, the formation of such channels was not followed by the massive outflow of the virus. Alternatively, the egress vacuoles inverted, that is, evaginated or bulged their content and formed membrane-coated sprouts with virus particles anchored to them (Figure 9B,C). The membranes seen at the outer surface of the cell originated mostly from the membrane of egress vacuoles, which explains the presence of cell cytosol in the sprouts. These rearrangements indicate a physical force, possibly osmotic, acting outward from the cell. The attachment of virus particles to the membranes seemed rather stable, and some enzyme activity or time would be needed to free the virus to the extracellular environment. For now, it could be only speculated about the molecular mechanisms behind these events. Calcium ions, entering the cytosol from extracellular space through the membrane-free borders and causing contraction and collapse of the cytoskeletal scaffold, and/or the osmotic pressure building up in the egress vacuoles might play a role in these processes.

After the virus egress, the Vero E6 cells were broken down to a critical state. Many cells lost most of their organelles (Figure 9D). Some cells remained with some intracellular structures present during virus replication, that is, DMVs, small mitochondria, and various vesicles. According to the overall picture of the infected cells, it would be reasonable to expect that after the virus egress, the cells are not viable and cannot resume their energetics and membranous organelles. However, exact evidence should be found in future studies.

### 3.6. SARS-CoV-2 Replication Dynamics and Autophagic Gene Expression

**Figure 10:**
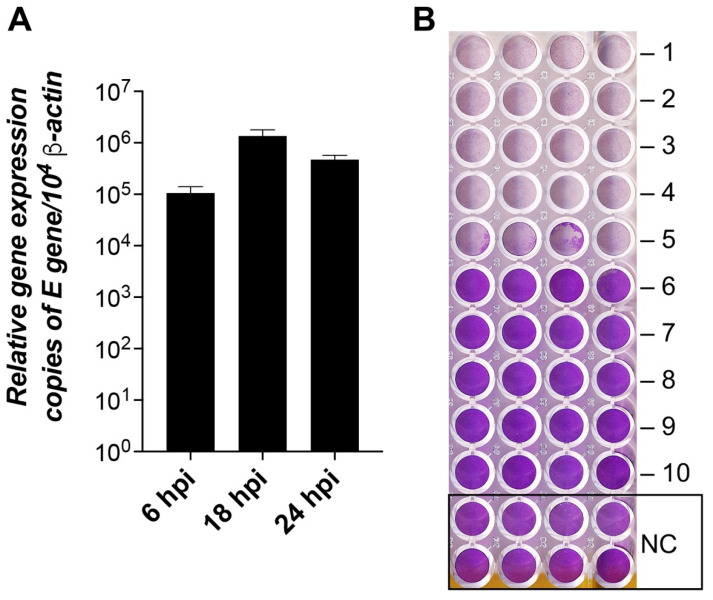
SARS-CoV-2 replication dynamics and infectivity in Vero E6 cells. (**A**) The columns show the mean of the viral E gene expression relative to the β-actin gene, n = 3. Error bars represent the standard error of the mean. hpi—hours post-infection. (**B**) TCID_50_ assay of cytopathic effect (CPE) of SARS-CoV-2 at 24 hpi. The plate shows cytopathic effects of the virus at dilutions -1 to -10 times and the control (NC) without the virus. The lower the violet signal intensity, the lower number of viable cells attached to the dish.

To ascertain the replication dynamics of SARS-CoV-2, we tested the Vero E6 cell lines for virus production at 6, 18, and 24 hpi (Figure 10A). The viral load was substantial already at 6 hpi (>90 K copies), reached the peak at 18 hpi (>1.2 M copies), and remained high at 24 hpi (>450 K copies). These data agree with previous estimates [5] and our TCID_50_ assay data (Figure 10B).

The transcript level of major genes of the autophagic pathway was quantified using cell host-specific qPCR arrays and compared with microscopic observations. Although the autophagic process is controlled by both the transcriptional and post-transcriptional mechanism [42], the qPCR arrays provide a simple and fast insight to compare changes in the activation of autophagy genes.

**Figure 11:**
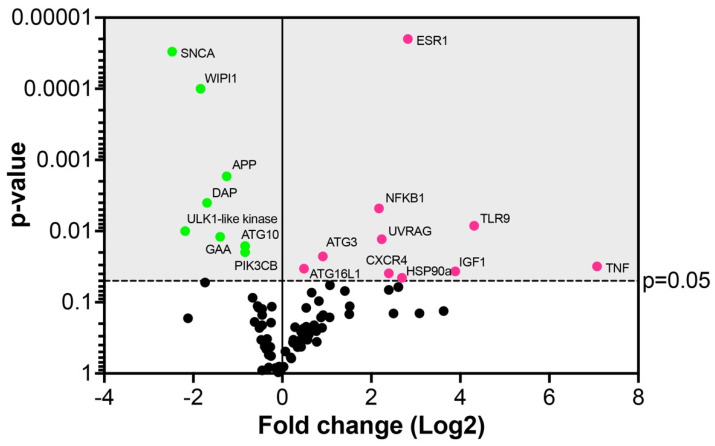
Autophagic gene array in Vero E6 cells 24 hpi with SARS-CoV-2. Dots in the volcano plot represent the mean of the fold-change (virus-infected vs. non-infected cells, n = 3) in the gene expression levels normalized using a panel of the internal housekeeping genes (red dots—upregulated genes, green dots—downregulated genes). The grey area indicates the cut-off level of *p* ≤ 0.05.

In virus-infected Vero E6 cells, out of 84 autophagic gene markers, 18 genes were significantly (*p* ≤ 0.05) fold regulated (Figure 11 and Table S1). Ten gene markers were upregulated (11.9%), whereas eight genes were downregulated (9.5%) (Figure 11 and Table S1). Among the upregulated genes, the highest expression levels exhibited the genes TNF, TLR9, IGF1, ESR1, CXCR4, and NFKB1 encoding the molecules primarily involved in the regulation of the immune responses or transcriptional machinery triggered by infecting viruses [43,44,45,46,47,48,49] (Figure 11 and Table S1).

Among the upregulated genes involved in autophagosome formation were UVRAG, ATG3, and ATG16L1. A component of the autophagy pathway, the UVRAG (UV radiation resistance-associated protein) gene, was significantly upregulated 4.7-fold in the virus-infected cells (Figure 11 and Table S1). The UVRAG represents a target for the virus to enhance its replication, as previously reported for the hepatitis C virus, which stimulated the maturation of autophagosomes [50]. The slightly yet significantly upregulated autophagy-related genes, ATG3 and ATG16L1, participate in lipidation and membrane association of LC3 which is crucial for the formation of autophagic vesicles, such as phagophores during coronavirus infection [15]. This is in line with our study describing the accumulation of phagophores in virus-infected Vero E6 cells, underscoring a role in virus replication.

As the number of genes significantly upregulated by SARS-CoV-2 was rather limited in this study, we considered it worth mentioning a few genes with borderline statistical significance to further correlations with our ultrastructure data. The upregulation of the unc51-like kinase (ULK2) at the 6.1-fold higher level (*p* = 0.061) in infected relative to uninfected cells suggests the proviral role of autophagy. This data can be correlated with electron microscopic images showing the formation of phagophores induced by the viral infection. The ULK2 is known to be activated upon induction of autophagy and to form a complex with ATG13 recruited to the membrane at the site of phagophore nucleation [19]. In addition, up to a 6.1-fold increase in the gene expression indicates that SARS-CoV-2 induces this step to augment the maturation of autophagosomes, probably to enhance viral replication, which is obvious from the electron microscopic observations. The ATG4A, upregulated by 2.1-fold (*p* = 0.057), is a cysteine protease interacting with proteins of the ATG8 family to mediate the insertion of the complex into the autophagosomal membranes, a step necessary for autophagy [51] and likely for the virus to boost the production of replication loci.

Among the downregulated genes, the highest level of downregulation (−5.5-fold) was detected for SNCA, alpha-synuclein (Figure 11 and Table S1). The protein is primarily found in neural tissue, which participates in synaptic vesicle exocytosis, modulates ER stress signaling, and suppresses the replication and growth of flavivirus [52]. The second most downregulated gene (−4.5-fold) was an ULK1-like kinase, which is activated at the autophagosome formation site and reportedly targeted by the papain-like protease of SARS-CoV-2 to suppress host autophagy [53]. The WD repeat domain phosphoinositide-interacting protein 1 (WIPI1, downregulated by −3.5-fold), as well as ATG10 and PIK3CB (both downregulated by −1.7-fold), are usually also found at the autophagosome formation site and incorporated into the autophagosomal membrane [19]. For increased autophagy, a significant −3.26-fold downregulation of the gene encoding death-associated protein 1 (DAP1) in virus-infected cells was particularly relevant. DAP1 is known to negatively regulate the autophagic process as its knockdown accelerates autophagosome accumulation and enhances autophagic activity in general [54]. Therefore, the downregulation of these genes may postpone the autophagosomal maturation process by the SARS-CoV-2 virus to utilize phagosomes for its replication.

The pro-apoptotic gene BCL-2, involved in mitochondrial turnover and autophagy, was downregulated by −3.3-fold at a borderline *p*-value (0.052). This might avert the advance of apoptosis as according to the previous studies, the BCL-2 was also downregulated by the viable and the inactivated coronaviruses, which indicates that BCL-2 is not required for virus replication [55].

## 4. Conclusions

The observations presented in this study show a high degree of similarity of the effect of SARS-CoV-2 on cell pathology with other viruses of the *Coronaviridae* family. Specifically, the formation of the virus replication complexes by transforming Golgi and endoplasmic reticulum membrane systems to assemble the nucleocapsids synthesized in the cytosol with the membrane envelope and its proteins. Moreover, as we showed, the transformation of Vero E6 cells by the SARS-CoV-2 also involves mitochondria that undergo extensive fission and membrane damage under the virus action. These modified mitochondria interact closely with phagophores and phagosomes during the period of virus maturation, with the evident assistance of lipid droplets. The activation of autophagy-like processes by virus infection was also obvious, however, at odds with the canonical autophagy. The autophagy process was modified to the extent that instead of protecting the cell from the pathogen, it formed a substrate for virus maturation and egress. As the autophagy-associated gene expression analysis revealed, some genes of the autophagy pathway were substantially upregulated and some were downregulated, but the majority of genes were regulated only weakly. The outcome of these virus-orchestrated processes in the susceptible cells was a massive release of virus particles to the extracellular space, leaving the cell remains in a state not compatible with life.

## Data Availability

Not applicable.

## Supplementary Materials

The following supporting information can be downloaded at: https://www.mdpi.com/article/10.3390/pathogens11121535/s1.

## References

[1] Siddell S., Wege H., ter Meulen V. (1982). The Structure and Replication of Coronaviruses. Curr. Top. Microbiol. Immunol..

[2] Klein S., Cortese M., Winter S.L., Wachsmuth-Melm M., Neufeldt C.J., Cerikan B., Stanifer M.L., Boulant S., Bartenschlager R., Chlanda P. (2020). SARS-CoV-2 Structure and Replication Characterized by in Situ Cryo-Electron Tomography. Nat. Commun..

[3] Caldas L.A., Carneiro F.A., Monteiro F.L., Augusto I., Higa L.M., Miranda K., Tanuri A., de Souza W. (2021). Intracellular Host Cell Membrane Remodelling Induced by SARS-CoV-2 Infection in Vitro. Biol. Cell.

[4] Hoffmann M., Kleine-Weber H., Pöhlmann S. (2020). A Multibasic Cleavage Site in the Spike Protein of SARS-CoV-2 Is Essential for Infection of Human Lung Cells. Mol. Cell.

[5] Hoffmann M., Kleine-Weber H., Schroeder S., Krüger N., Herrler T., Erichsen S., Schiergens T.S., Herrler G., Wu N.H., Nitsche A. (2020). SARS-CoV-2 Cell Entry Depends on ACE2 and TMPRSS2 and Is Blocked by a Clinically Proven Protease Inhibitor. Cell.

[6] De Wilde A.H., Snijder E.J., Kikkert M., Van Hemert M.J., De Wilde A.H., Snijder Á.E.J., Kikkert Á.M., Van Hemert Á.M.J. (2017). Host Factors in Coronavirus Replication. Curr. Top. Microbiol. Immunol..

[7] de Breyne S., Vindry C., Guillin O., Condé L., Mure F., Gruffat H., Chavatte L., Ohlmann T. (2020). Translational Control of Coronaviruses. Nucleic Acids Res..

[8] Mihelc E.M., Baker S.C., Lanman J.K. (2021). Coronavirus Infection Induces Progressive Restructuring of the Endoplasmic Reticulum Involving the Formation and Degradation of Double Membrane Vesicles. Virology.

[9] Lavi E., Wang Q., Weiss S.R., Gonatas N.K. (1996). Syncytia Formation Induced by Coronavirus Infection Is Associated with Fragmentation and Rearrangement of the Golgi Apparatus. Virology.

[10] Knoops K., Kikkert M., Worm S.H.E.V.D., Zevenhoven-Dobbe J.C., Meer Y.V.D., Koster A.J., Mommaas A.M., Snijder E.J. (2008). SARS-Coronavirus Replication Is Supported by a Reticulovesicular Network of Modified Endoplasmic Reticulum. PLoS Biol..

[11] Wong N.A., Saier M.H. (2021). The Sars-Coronavirus Infection Cycle: A Survey of Viral Membrane Proteins, Their Functional Interactions and Pathogenesis. Int. J. Mol. Sci..

[12] Ducatelle R., Hoorens J. (1984). Significance of Lysosomes in the Morphogenesis of Coronaviruses. Arch. Virol..

[13] Ghosh S., Dellibovi-Ragheb T.A., Kerviel A., Pak E., Qiu Q., Fisher M., Takvorian P.M., Bleck C., Hsu V.W., Fehr A.R. (2020). β-Coronaviruses Use Lysosomes for Egress Instead of the Biosynthetic Secretory Pathway. Cell.

[14] Ahmad L., Mostowy S., Sancho-Shimizu V. (2018). Autophagy-Virus Interplay: From Cell Biology to Human Disease. Front. Cell Dev. Biol..

[15] Miller K., McGrath M.E., Hu Z., Ariannejad S., Weston S., Frieman M., Jackson W.T. (2020). Coronavirus Interactions with the Cellular Autophagy Machinery. Autophagy.

[16] Zhao Z., Lu K., Mao B., Liu S., Trilling M., Huang A., Lu M., Lin Y. (2021). The Interplay between Emerging Human Coronavirus Infections and Autophagy. Emerg. Microbes Infect..

[17] Zhang L., Qin Y., Chen M. (2018). Viral Strategies for Triggering and Manipulating Mitophagy. Autophagy.

[18] Shi C.-S., Qi H.-Y., Boularan C., Huang N.-N., Abu-Asab M., Shelhamer J.H., Kehrl J.H. (2014). SARS-Coronavirus Open Reading Frame-9b Suppresses Innate Immunity by Targeting Mitochondria and the MAVS/TRAF3/TRAF6 Signalosome. J. Immunol..

[19] Carlsson S.R., Simonsen A. (2015). Membrane Dynamics in Autophagosome Biogenesis. J. Cell Sci..

[20] Delorme-Axford E., Klionsky D.J. (2020). Highlights in the Fight against COVID-19: Does Autophagy Play a Role in SARS-CoV-2 Infection?. Autophagy.

[21] Maier H.J., Britton P. (2012). Involvement of Autophagy in Coronavirus Replication. Viruses.

[22] Schneider M., Ackermann K., Stuart M., Wex C., Protzer U., Schätzl H.M., Gilch S. (2012). Severe Acute Respiratory Syndrome Coronavirus Replication Is Severely Impaired by MG132 Due to Proteasome-Independent Inhibition of M-Calpain. J. Virol..

[23] Yang N., Shen H.M. (2020). Targeting the Endocytic Pathway and Autophagy Process as a Novel Therapeutic Strategy in COVID-19. Int. J. Biol. Sci..

[24] Gassen N.C., Niemeyer D., Muth D., Corman V.M., Martinelli S., Gassen A., Hafner K., Papies J., Mösbauer K., Zellner A. (2019). SKP2 Attenuates Autophagy through Beclin1-Ubiquitination and Its Inhibition Reduces MERS-Coronavirus Infection. Nat. Commun..

[25] Ramirez S., Fernandez-Antunez C., Galli A., Underwood A., Pham L.V., Ryberg L.A., Feng S., Pedersen M.S., Mikkelsen L.S., Belouzard S. (2021). Overcoming Culture Restriction for SARS-CoV-2 in Human Cells Facilitates the Screening of Compounds Inhibiting Viral Replication. Antimicrob. Agents Chemother..

[26] Corman V.M., Landt O., Kaiser M., Molenkamp R., Meijer A., Chu D.K.W., Bleicker T., Brünink S., Schneider J., Schmidt M.L. (2020). Detection of 2019 Novel Coronavirus (2019-NCoV) by Real-Time RT-PCR. Eurosurveillance.

[27] Ge L., Schekman R. (2014). The ER-Golgi Intermediate Compartment Feeds the Phagophore Membrane. Autophagy.

[28] Hagemeijer M.C., Verheije M.H., Ulasli M., Shaltiël I.A., de Vries L.A., Reggiori F., Rottier P.J.M., de Haan C.A.M. (2010). Dynamics of Coronavirus Replication-Transcription Complexes. J. Virol..

[29] V’kovski P., Kratzel A., Steiner S., Stalder H., Thiel V. (2021). Coronavirus Biology and Replication: Implications for SARS-CoV-2. Nat. Rev. Microbiol..

[30] Farkash E.A., Wilson A.M., Jentzen J.M. (2020). Ultrastructural Evidence for Direct Renal Infection with SARS-CoV-2. J. Am. Soc. Nephrol..

[31] Ogando N.S., Dalebout T.J., Zevenhoven-Dobbe J.C., Limpens R.W.A.L., van der Meer Y., Caly L., Druce J., de Vries J.J.C., Kikkert M., Bárcena M. (2020). SARS-Coronavirus-2 Replication in Vero E6 Cells: Replication Kinetics, Rapid Adaptation and Cytopathology. J. Gen. Virol..

[32] Gatti P., Ilamathi H.S., Todkar K., Germain M. (2020). Mitochondria Targeted Viral Replication and Survival Strategies—Prospective on SARS-CoV-2. Front. Pharmacol..

[33] Yamashita S.I., Jin X., Furukawa K., Hamasaki M., Nezu A., Otera H., Saigusa T., Yoshimori T., Sakai Y., Mihara K. (2016). Mitochondrial Division Occurs Concurrently with Autophagosome Formation but Independently of Drp1 during Mitophagy. J. Cell Biol..

[34] Hailey D.W., Rambold A.S., Satpute-Krishnan P., Mitra K., Sougrat R., Kim P.K., Lippincott-Schwartz J. (2010). Mitochondria Supply Membranes for Autophagosome Biogenesis during Starvation. Cell.

[35] Biazik J., Ylä-Anttila P., Vihinen H., Jokitalo E., Eskelinen E.L. (2015). Ultrastructural Relationship of the Phagophore with Surrounding Organelles. Autophagy.

[36] Cook K.L., Soto-Pantoja D.R., Abu-Asab M., Clarke P.A.G., Roberts D.D., Clarke R. (2014). Mitochondria Directly Donate Their Membrane to Form Autophagosomes during a Novel Mechanism of Parkin-Associated Mitophagy. Cell Biosci..

[37] Saka H.A., Valdivia R. (2012). Emerging Roles for Lipid Droplets in Immunity and Host-Pathogen Interactions. Annu. Rev. Cell Dev. Biol..

[38] Viktorova E.G., Nchoutmboube J.A., Ford-Siltz L.A., Iverson E., Belov G.A. (2018). Phospholipid Synthesis Fueled by Lipid Droplets Drives the Structural Development of Poliovirus Replication Organelles. PLoS Pathog..

[39] Zhang Z., He G., Filipowicz N.A., Randall G., Belov G.A., Kopek B.G., Wang X. (2019). Host Lipids in Positive-Strand RNA Virus Genome Replication. Front. Microbiol..

[40] Pagliari F., Marafioti M.G., Genard G., Candeloro P., Viglietto G., Seco J., Tirinato L. (2020). SsRNA Virus and Host Lipid Rearrangements: Is There a Role for Lipid Droplets in SARS-CoV-2 Infection?. Front. Mol. Biosci..

[41] Stertz S., Reichelt M., Spiegel M., Kuri T., Martínez-Sobrido L., García-Sastre A., Weber F., Kochs G. (2007). The Intracellular Sites of Early Replication and Budding of SARS-Coronavirus. Virology.

[42] di Malta C., Cinque L., Settembre C. (2019). Transcriptional Regulation of Autophagy: Mechanisms and Diseases. Front. Cell Dev. Biol..

[43] Robinson P.C., Liew D.F.L., Liew J.W., Monaco C., Richards D., Shivakumar S., Tanner H.L., Feldmann M. (2020). The Potential for Repurposing Anti-TNF as a Therapy for the Treatment of COVID-19. Med.

[44] Sato A., Linehan M.M., Iwasaki A. (2006). Dual Recognition of Herpes Simplex Viruses by TLR2 and TLR9 in Dendritic Cells. Proc. Natl. Acad. Sci. USA.

[45] Sainz B., Mossel E.C., Peters C.J., Garry R.F. (2004). Interferon-Beta and Interferon-Gamma Synergistically Inhibit the Replication of Severe Acute Respiratory Syndrome-Associated Coronavirus (SARS-CoV). Virology.

[46] Józefiak A., Larska M., Pomorska-Mól M., Ruszkowski J.J. (2021). The IGF-1 Signaling Pathway in Viral Infections. Viruses.

[47] Shin G.C., Kang H.S., Lee A.R., Kim K.H. (2016). Hepatitis B Virus–Triggered Autophagy Targets TNFRSF10B/Death Receptor 5 for Degradation to Limit TNFSF10/TRAIL Response. Autophagy.

[48] Espert L., Denizot M., Grimaldi M., Robert-Hebmann V., Gay B., Varbanov M., Codogno P., Biard-Piechaczyk M. (2006). Autophagy Is Involved in T Cell Death after Binding of HIV-1 Envelope Proteins to CXCR4. J. Clin. Investig..

[49] Das B., Dobrowolski C., Luttge B., Valadkhan S., Chomont N., Johnston R., Bacchetti P., Hoh R., Gandhi M., Deeks S.G. (2018). Estrogen Receptor-1 Is a Key Regulator of HIV-1 Latency That Imparts Gender-Specific Restrictions on the Latent Reservoir. Proc. Natl. Acad. Sci. USA.

[50] Wang L., Tian Y., Ou J.-H.J. (2015). HCV Induces the Expression of Rubicon and UVRAG to Temporally Regulate the Maturation of Autophagosomes and Viral Replication. PLoS Pathog..

[51] Skytte Rasmussen M., Mouilleron S., Kumar Shrestha B., Wirth M., Lee R., Bowitz Larsen K., Abudu Princely Y., O’Reilly N., Sjøttem E., Tooze S.A. (2017). ATG4B Contains a C-Terminal LIR Motif Important for Binding and Efficient Cleavage of Mammalian Orthologs of Yeast Atg8. Autophagy.

[52] Beatman E.L., Massey A., Shives K.D., Burrack K.S., Chamanian M., Morrison T.E., Beckham J.D. (2016). Alpha-Synuclein Expression Restricts RNA Viral Infections in the Brain. J. Virol..

[53] Mohamud Y., Xue Y.C., Liu H., Ng C.S., Bahreyni A., Jan E., Luo H. (2021). The Papain-like Protease of Coronaviruses Cleaves ULK1 to Disrupt Host Autophagy. Biochem. Biophys. Res. Commun..

[54] Koren I., Reem E., Kimchi A. (2010). DAP1, a Novel Substrate of MTOR, Negatively Regulates Autophagy. Curr. Biol..

[55] Mehrzadi S., Karimi M.Y., Fatemi A., Reiter R.J., Hosseinzadeh A. (2021). SARS-CoV-2 and Other Coronaviruses Negatively Influence Mitochondrial Quality Control: Beneficial Effects of Melatonin. Pharmacol. Ther..

